# Supplementary material for: The interindividual variability of multimodal brain connectivity maintains spatial heterogeneity and relates to tissue microstructure
Source: Commun Biol. 2022 Sep 23;5:1007. doi: 10.1038/s42003-022-03974-w (PMC9508245; doi:10.1038/s42003-022-03974-w)
Supplement: Supplementary file 2 — Supplementary Information [file 42003_2022_3974_MOESM2_ESM.pdf]

The interindividual variability of multimodal brain connectivity maintains spatial heterogeneity and relates to tissue microstructure

Supplementary information

Esin Karahan, Luke Tait, Ruoguang Si, Ayşegül Özkan, Maciek J Szul, Kim S. Graham, Andrew D. Lawrence, Jiaxiang Zhang

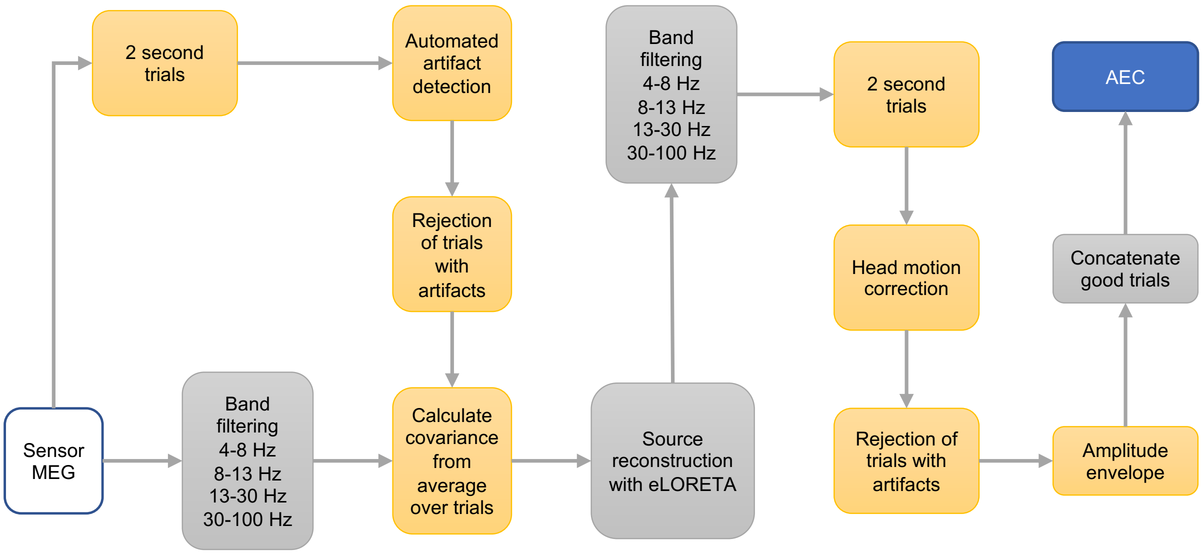

Supplementary Fig. 1: MEG pre-processing pipeline. For source reconstruction, the down-sampled HCP-MMP atlas[1] was used.

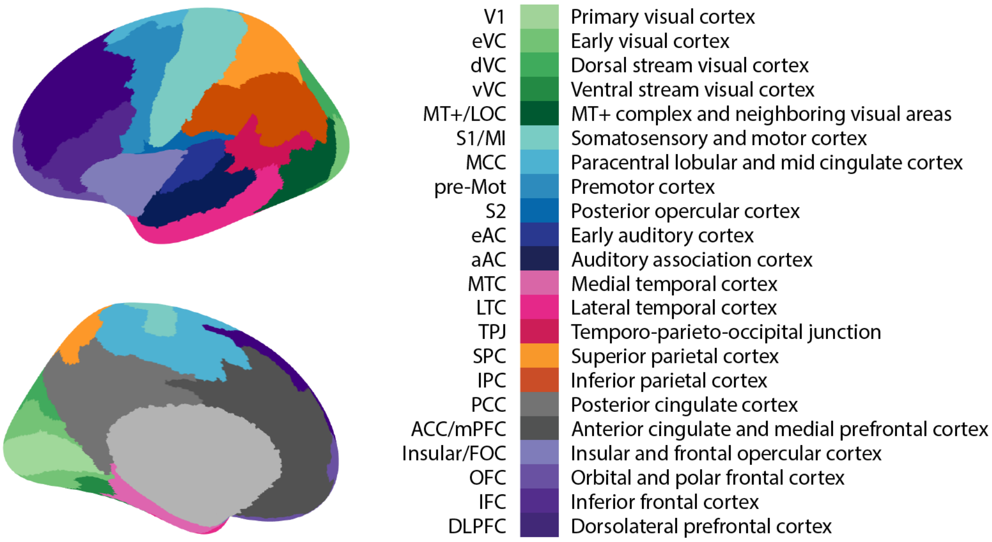

Supplementary Fig. 2: 22 cortical clusters defined in the HCP-MMP atlas[2]. Clusters close to each other are rendered in similar colors. The full name in the original HCP-MMP atlas and the acronym of each cluster were shown. For the cluster of MT+ and neighbouring visual areas, we used the acronym MT+/LOC as the neighbouring areas mainly include the lateral occipital cortex (LOC). For the cluster of posterior opercular cortex, we used the acronym S2, as the second somatosensory area makes most of this cluster.

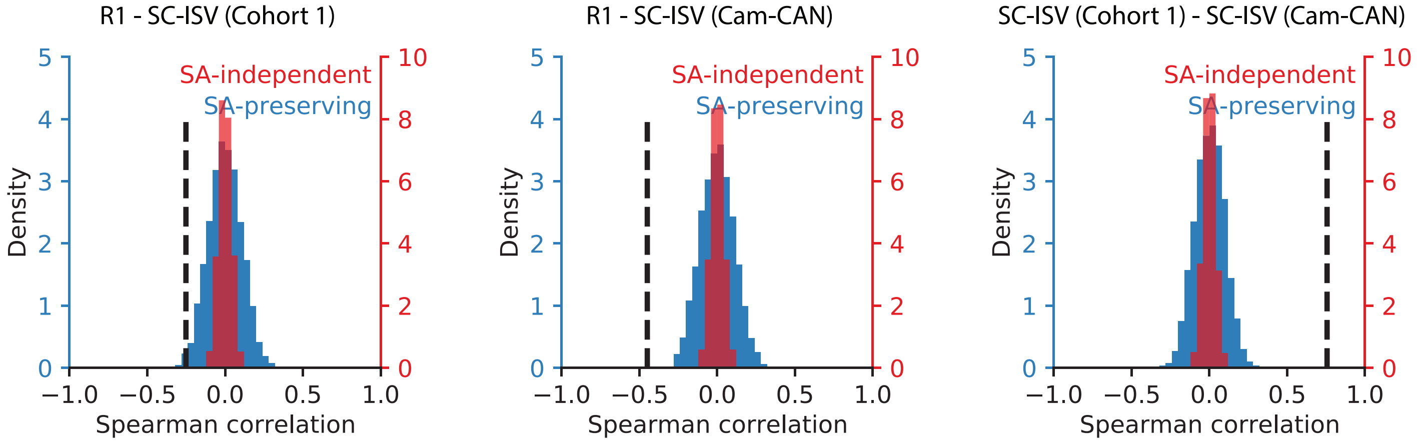

Supplementary Fig. 3: Null distributions and observed Spearman correlation coefficients relate to SC-ISV maps. Left: correlations between the R1 map and Cohort 1 SC-ISV. Middle: correlations between the R1 map and Cam-CAN SC-ISV. Right: correlations between Cohort 1 and Cam-CAN SC-ISV). SA-preserving null distributions (in red) were obtained from correlations with 5,000 surrogate maps with the same spatial autocorrelation as in the empirical brain maps. SA-independent null distributions (in blue) were obtained from correlations with 5,000 iterations of randomly shuffled values. Dashed vertical line indicates the empirical Spearman's correlation coefficient.

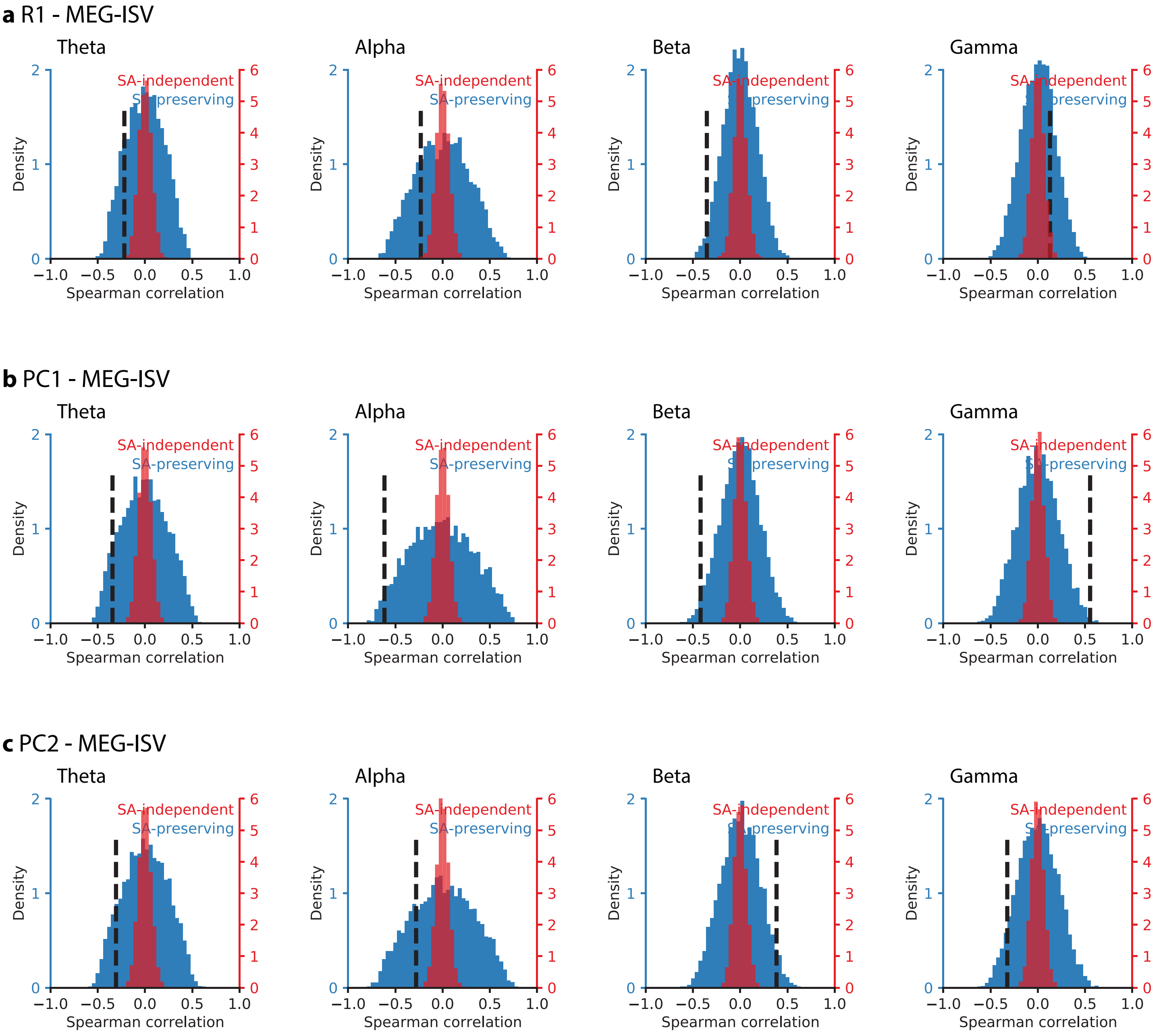

Supplementary Fig. 4: Null distributions and observed Spearman correlation coefficients relate to MEG-ISV maps. a Correlations between the R1 map and MEG-ISV. **b** Correlations between the PC1 map and MEG-ISV. **c** Correlations between the PC2 map and MEG-ISV. Each panel showed results from all four MEG frequency bands. SA-preserving null distributions (in red) were obtained from correlations with 5,000 surrogate maps with the same spatial autocorrelation as in the empirical brain maps. SA-independent null distributions (in blue) were obtained from correlations with 5,000 iterations of randomly shuffled values. Dashed vertical line indicates the empirical Spearman's correlation coefficient.

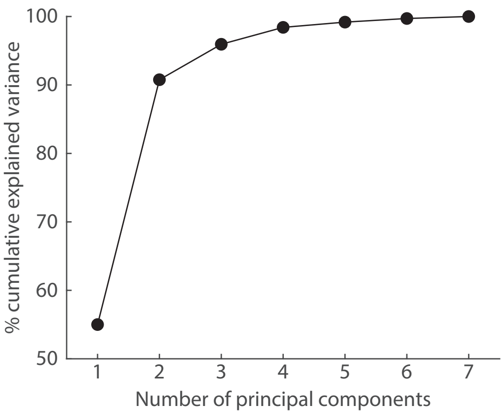

Supplementary Fig. 5: The cumulative explained variance of principal components from the PCA analysis of 7 microstructural metrics across participants and connections.

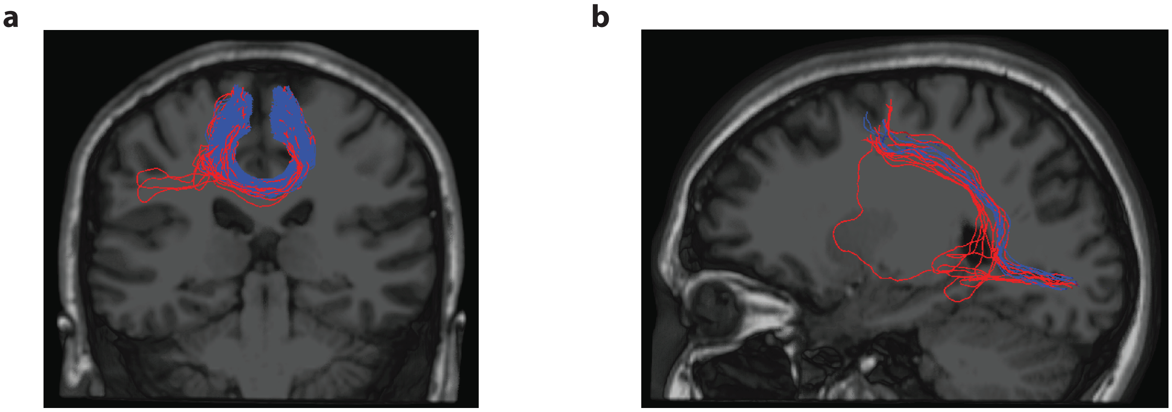

Supplementary Fig. 6: Demonstration of the streamline trimming procedure on a sample subject's tractography result. a Cross-hemispheric streamlines connecting the left and right SCEF (supplementary and cingulate eye field). **b** Intra hemispheric streamlines connecting the left V1 to the left i6-8 (Inferior 6-8 Transitional Area). In both panels, red streamlines are the ones rejected by the trimming procedure, and blue streamlines are the ones survived.

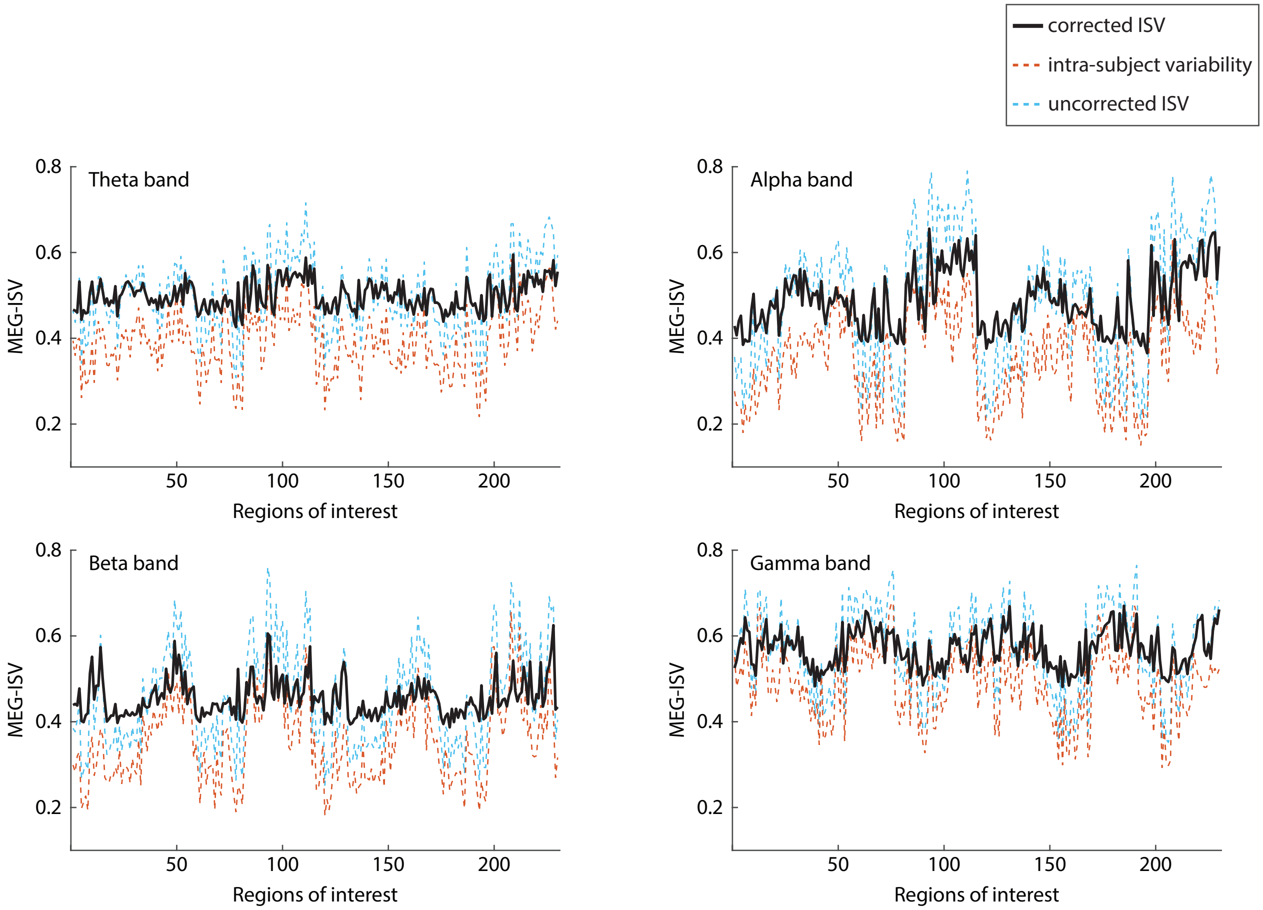

Supplementary Fig. 7: MEG-ISV before and after correction for intra-subject variability. For each frequency band, correction was performed on the ISV for each pair of subjects. The plots illustrate the ISV value of each cortical ROIs from the downsampled HCP-MMP atlas.

Supplementary References

1. Tait, L., Özkan, A., Szul, M. J. & Zhang, J. A systematic evaluation of source reconstruction of resting MEG of the human brain with a new high-resolution atlas: Performance, precision, and parcellation. *Hum. Brain Mapp.* **42**, 4685–4707 (2021).

2. Glasser, M. F. *et al.* A multi-modal parcellation of human cerebral cortex. *Nature* **536**, 171–178 (2016).
