# Supplementary material for: The interindividual variability of multimodal brain connectivity maintains spatial heterogeneity and relates to tissue microstructure
Source: Commun Biol. 2022 Sep 23;5:1007. doi: 10.1038/s42003-022-03974-w (PMC9508245; doi:10.1038/s42003-022-03974-w)
Supplement: Supplementary file 3 — Reporting summary [file 42003_2022_3974_MOESM3_ESM.pdf]

nature portfolio

Corresponding author(s): Jiaxiang Zhang
COMMSBIO-21-3067B

Last updated by author(s): Aug 6, 2022

Reporting Summary

Nature Portfolio wishes to improve the reproducibility of the work that we publish. This form provides structure for consistency and transparency in reporting. For further information on Nature Portfolio policies, see our Editorial Policies and the Editorial Policy Checklist.

Statistics

For all statistical analyses, confirm that the following items are present in the figure legend, table legend, main text, or Methods section.

n/a	Confirmed	
☐	☒	The exact sample size (*n*) for each experimental group/condition, given as a discrete number and unit of measurement
☐	☒	A statement on whether measurements were taken from distinct samples or whether the same sample was measured repeatedly
☐	☒	The statistical test(s) used AND whether they are one- or two-sided<br>*Only common tests should be described solely by name; describe more complex techniques in the Methods section.*
☐	☒	A description of all covariates tested
☐	☒	A description of any assumptions or corrections, such as tests of normality and adjustment for multiple comparisons
☐	☒	A full description of the statistical parameters including central tendency (e.g. means) or other basic estimates (e.g. regression coefficient) AND variation (e.g. standard deviation) or associated estimates of uncertainty (e.g. confidence intervals)
☐	☒	For null hypothesis testing, the test statistic (e.g. *F*, *t*, *r*) with confidence intervals, effect sizes, degrees of freedom and *P* value noted<br>*Give P values as exact values whenever suitable.*
☒	☐	For Bayesian analysis, information on the choice of priors and Markov chain Monte Carlo settings
☒	☐	For hierarchical and complex designs, identification of the appropriate level for tests and full reporting of outcomes
☐	☒	Estimates of effect sizes (e.g. Cohen's *d*, Pearson's *r*), indicating how they were calculated

Our web collection on statistics for biologists contains articles on many of the points above.

Software and code

Policy information about availability of computer code

Data collection	MRI data were acquired from Siemens 3T Connectom MRI system and Siemens 7T Magnetom MRI scanner (Siemens Medical Systems, Germany). MEG recordings were acquired from a 275-channel CTF radial gradiometer system (CTF Systems, Canada).
Data analysis	Custom made scripts were developed in bash and MATLAB R2018b. Freesurfer version 5.3.0 was used to process T1-weighted MP-RAGE images. DWI data were processed with FSL version 6.0.1 and analyzed with MRTrix version 3. To create microstructural measures from DWI data, we used NODDI MATLAB Toolbox v1.0.1. MEG data pipeline was developed with custom MATLAB scripts built upon Fieldtrip toolbox for M/EEG analysis. MP2RAGE images acquired from 7T MRI were processed with Freesurfer version 6.0.1, FSL version 6.0.1, Java Image Science Toolkit, and the CBS High-Res Brain Processing tools of the MIPAV platform.

For manuscripts utilizing custom algorithms or software that are central to the research but not yet described in published literature, software must be made available to editors and reviewers. We strongly encourage code deposition in a community repository (e.g. GitHub). See the Nature Portfolio guidelines for submitting code & software for further information.

Data

Policy information about availability of data

All manuscripts must include a data availability statement. This statement should provide the following information, where applicable:

- Accession codes, unique identifiers, or web links for publicly available datasets
- A description of any restrictions on data availability
- For clinical datasets or third party data, please ensure that the statement adheres to our policy

Data availability: DWI, MEG and 7T MRI data that support the findings of this study and data from all analyses are available in OSF with the unique identifier [https://doi.org/10.17605/osf.io/rqj8a]. A part of data used in the preparation of this work were obtained from the Cam-CAN repository (http://www.mrc-cbu.cam.ac.uk/

datasets/camcan).

Code availability: The scripts that support the calculation of the ISV and statistical analyses used in this study are available in OSF with the unique identifier [https://doi.org/10.17605/osf.io/rqj8a].

Field-specific reporting

Please select the one below that is the best fit for your research. If you are not sure, read the appropriate sections before making your selection.

☒ Life sciences ☐ Behavioural & social sciences ☐ Ecological, evolutionary & environmental sciences

For a reference copy of the document with all sections, see nature.com/documents/nr-reporting-summary-flat.pdf

Life sciences study design

All studies must disclose on these points even when the disclosure is negative.

Sample size	This study did not perform power calculation but used sample-size comparable to or larger than previous studies. For calculating the intersubject variability of fMRI and DWI based connectome, previous studies used 25 (Mueller et al., 2013) or 9 (Chamberland et al., 2017) participants. Their calculation of intersubject variability of connectivity is consistent with that from larger cohorts (e.g., Mansour et al., 2021 and Demirtas et al., 2019), suggesting that, for young healthy volunteers, the intersubject variability can be reliability quantified from <30 participants. The current study collected multimodal (DWI and MEG) data from 51 participants in Cohort 1.<br><br>For the 7T R1 measure in Cohort 2, previous research (Marques et al., 2017, Shams et al., 2019 and Haast et al., 2016) have identified robust cortical R1 measures using a small sample size (N~10), because R1 does not exhibit substantial variability between young healthy participants. Current study acquired R1 measures from 35 healthy participants. Our group R1 maps replicated previous studies that used a smaller number of samples and also other studies that quantified cortical myelin content with different MRI sequences (Glasser et al., 2011 and Glasser et al., 2014).
Data exclusions	51 participants from Cohort 1 completed diffusion MRI (dMRI), and 28 participants had MEG acquisitions. Data quality for anatomical MRI and dMRI data from Cohort 1 was checked with custom scripts for head motion, registration faults and EPI distortions. MEG data from all subjects were corrected for motion artifacts both in sensor and source level. None of the subjects had extreme motion artifacts.<br>For 7T MP2RAGE data, Two participants had distorted R1 images and were removed from subsequent analyses. We did not include lateral temporal cortex in R1 map calculations due to intensity artifacts in this region in all subjects.
Replication	Cortical myelin content maps derived from R1 images were consistent with Glasser et al., 2014, Lutti et al., 2014, Marques et al., 2017, Shams et al., 2019 and Haast et al., 2016.<br><br>To ensure the replicability of our findings, we have made the imaging data and scripts for individual variability analysis available online. Details are available in the data and code availability statements of the manuscript.
Randomization	This is not relevant to the current study because there were no separate experimental groups.
Blinding	Blinding is not performed since there were no experimental groups.

Reporting for specific materials, systems and methods

We require information from authors about some types of materials, experimental systems and methods used in many studies. Here, indicate whether each material, system or method listed is relevant to your study. If you are not sure if a list item applies to your research, read the appropriate section before selecting a response.

Materials & experimental systems

n/a	Involved in the study	
☒	☐	Antibodies
☒	☐	Eukaryotic cell lines
☒	☐	Palaeontology and archaeology
☒	☐	Animals and other organisms
☐	☒	Human research participants
☒	☐	Clinical data
☒	☐	Dual use research of concern

Methods

n/a	Involved in the study	
☒	☐	ChIP-seq
☒	☐	Flow cytometry
☐	☒	MRI-based neuroimaging

Human research participants

Policy information about studies involving human research participants

Population characteristics	86 healthy participants were recruited from a local participant panel consisted of undergraduate and postgraduate students.

Population characteristics	Cohort 1 included 51 participants (36 females, age range 18-28 years; mean age 21.20 +/- 2.74 s.d.). All participants in Cohort 1 underwent a 3T MRI session, and 28 participants in Cohort 1 completed two further MEG sessions. Cohort 2 included 35 participants (29 females, age range 18-35 years; mean age 21.22 +/- 3.5 years), and all completed a 7T MRI session. There was no significant difference in age (t(84)=-0.05, p=0.96) or gender (chi^2 = 1.69, p=0.19) between the two cohorts. No participant reported a history of neurological or psychiatric illness.
Recruitment	Participants were recruited from a local participant panel by online announcements through university.
Ethics oversight	The study was approved by the university's School of Psychology Research Ethics Committee. All participants gave written informed consent.

Note that full information on the approval of the study protocol must also be provided in the manuscript.

Magnetic resonance imaging

Experimental design

Design type	Resting state MEG, microstructural analysis and tractography with diffusion MRI, cortical myelin content quantification with MP2RAGE imaging sequence
Design specifications	For MEG resting-state sessions, participants were instructed to rest with their eyes open and fixate on a red dot with a grey background, presented through a back projector. Each recording session lasted approximately 8 minutes.
Behavioral performance measures	No tasks were performed during scanning, so behavioral performance measures were not recorded.

Acquisition

Imaging type(s)	Structural, diffusion, T1 relaxation rate (R1), functional (MEG)
Field strength	3T and 7T
Sequence & imaging parameters	Whole-brain, multi-shell, diffusion-weighted images (DWI) were acquired using a Siemens 3T Connectom MRI scanner with a strong gradient of 300mT/m (Siemens Medical Systems). The spin-echo echoplanar imaging (EPI) pulse sequence used a HARDI protocol (echo time 59 ms, repetition time 3000 ms, voxel size 2x2x2 mm).<br><br>Participants also underwent high-resolution T1-weighted magnetization prepared rapid gradient echo scanning (MP-RAGE: echo time 3.06 ms; repetition time 2250 ms sequence, flip angle 9°, field of view=256x256 mm, acquisition matrix 256x256, voxel size 1x1x1 mm).<br><br>Whole-brain, high-resolution and high-field structural imaging were acquired from all participants in Cohort 2 on a Siemens 7T Magnetom MRI scanner (Siemens Medical Systems, Germany) using a 32 channel head coil (Nova Medical, USA). The MP2RAGE sequence was used which included two MPRAGE acquisitions with different flip angles and inversion times (echo time 2.68 ms, repetition time 6000 ms, first inversion time 800 ms, second inversion time 2700 ms, first flip angle 7, second flip angle 5, voxel size 0.65x0.65x0.65 mm3). To correct for the RF transmit field B+1, whole-brain B+1 images were acquired using the saturation-prepared with 2 rapid gradient echoes (SA2RAGE) sequence (echo time 1.16 ms, repetition time 2400 ms, first inversion time 540 ms, second inversion time 1800 ms, first flip angle 4, second flip angle 11, voxel size 3.25x3.25x3 mm3).
Area of acquisition	Whole brain

Diffusion MRI ☒ Used ☐ Not used

Parameters	Diffusion sensitizing gradients were applied in 20 isotropic directions at b-values of 200 and 500 s/mm2, in 30 isotropic directions at a b-value of 1200 s/mm2 and in 61 isotropic directions at b-values of 2400, 4000, 6000 s/mm2. Thirteen volumes with no diffusion weighting (b = 0 s/mm2) interleaved across the sequence were also acquired. To correct for susceptibility induced distortions, three images at b=0 s/mm2 and 30 diffusion directions at b = 1200 s/mm2 were acquired with the opposite phase encoding direction.

Preprocessing

Preprocessing software	FMRIB's Software Library (FSL), v6.0.1<br>MRtrix3<br>Freesurfer v5.3.0 and v6.0.1<br>MIPAV<br>Java Image Science Toolkit<br>NODDI MATLAB Toolbox v1.0.1<br>Fieldtrip
Normalization	No normalization was performed. Diffusion MRI and R1 data were analyzed in subject's native space.
Normalization template	Data were not normalized. However, Human Connectome Project Multi-Modal Parcellation (HCP-MMP) atlas (Glasser et al., 2016) was used for parcellation of cortical surface by registering the atlas to native space.
Noise and artifact removal	Diffusion MRI data were denoised using the MP-PCA noise estimation function in MRTrix3, were corrected for drift (Vos et

Noise and artifact removal | al., 2017), were corrected for susceptibility induced distortions, eddy currents and head motion using FSL. Diffusion MRI data were corrected for gradient nonlinearity and Gibbs ringing artefacts (Kellner et al., 2016). After region-to-region tractography, outlier streamlines were eliminated by using k-means clustering.

Volume censoring | Diffusion MRI data were corrected for motion.

Statistical modeling & inference

Model type and settings | Principal component analysis, Pearson and Spearman rank correlation

Effect(s) tested | There were no stimulus/task conditions, therefore no effects were tested.

Specify type of analysis: ☐ Whole brain ☒ ROI-based ☐ Both

Anatomical location(s) | Anatomical locations were determined by Human Connectome Project Multi-Modal Parcellation (HCP-MMP) atlas (Glasser et al., 2016).

Statistic type for inference (See Eklund et al. 2016) | We performed statistics on clusters and region of interests defined by HCP-MMP atlas.

Correction | Confidence intervals for clusters of structural and functional intersubject variability were calculated from 5000 bootstrap samples.
To correct for multiple comparisons across the 22 cortical clusters, we used permutation based maximum statistic with 10,000 permutations to control for the family-wise error (FWE) rate.
To assess the correlation between structural, functional intersubject variability and R1 value and white matter microstructure we used Spearman rank correlations and obtained significance with permutation testing.

Models & analysis

n/a	Involved in the study
☐	☒ Functional and/or effective connectivity
☒	☐ Graph analysis
☐	☒ Multivariate modeling or predictive analysis

Functional and/or effective connectivity | MEG based functional connectivity was constructed by using amplitude envelope correlation (AEC) within two frequency bands. Prior to AEC, leakage correction was performed with multivariate orthogonalization (Colclough et al., 2016). Pearson correlation between two pairs of ROIs was calculated. We thresholded MEG connectivity matrices by thresholding to retain strongest 75% connections which obtained from graph measures (Achard 2007).

Multivariate modeling and predictive analysis | All microstructural measures were sampled along the streamlines by using tcksample function from MRTrix. We took the median value of measures along and across streamlines to characterize microstructural properties of region to region connections. By this way, we obtained a 664 × 664 × 7 maxtrix for each participant, representing microstructrual measues of structural connectivity. We converted microstructural measures to z-score per subject to avoid scale differences between measures and participants.

We used principal component analysis to reduce the dimensionality of the microstructural measures. PCA was applied to the seven microstructural measures across participants and structural connections. We retained first two principal components which explained >90% variance in the data.
